# Prevalence and Characteristics of Manipulative Design in Mobile Applications Used by Children

**DOI:** 10.1001/jamanetworkopen.2022.17641

**Published:** 2022-06-17

**Authors:** Jenny Radesky, Alexis Hiniker, Caroline McLaren, Eliz Akgun, Alexandria Schaller, Heidi M. Weeks, Scott Campbell, Ashley N. Gearhardt

**Affiliations:** 1 Department of Pediatrics, University of Michigan Medical School, Ann Arbor; 2 University of Washington Information School, Seattle; 3 University of Michigan School of Public Health, Ann Arbor; 4 Department of Communication and Media Studies, University of Michigan, Ann Arbor; 5 Department of Psychology, University of Michigan, Ann Arbor

## Abstract

**Importance:**

Manipulative design features (known as dark patterns) are common in video games and adult-directed technologies, but their prevalence in children’s interactive media has not been described.

**Objectives:**

To develop a reliable coding scheme for gathering data on manipulative digital designs, describe their prevalence within apps used by a community-based sample of young children, and test hypotheses about associations of manipulative design features with socioeconomic status (SES).

**Design, Setting, and Participants:**

This cross-sectional study of a convenience sample of parents of children aged 3 to 5 years was conducted online. Eligible parents were legal guardians of a 3-to-5-year-old child, lived with their child at least 5 days per week, understood English, and were part of a family that owned at least 1 Android or iOS tablet or smartphone. For each participant, the 3 apps used for the longest duration by children with their own mobile devices were downloaded, played, and coded. Data were analyzed between April and August 2021.

**Exposures:**

Child SES, operationalized as parent educational attainment and household income-to-needs ratio.

**Main Outcomes and Measures:**

Researchers assigned each child a prevalence score for manipulative design features (overall, gameplay pressure, purchase pressure, and advertisement viewing pressure) within the apps children played.

**Results:**

Of 160 children in the sample, mean (SD) age was 4.0 (0.6) years; 120 children (75.0%) were non-Hispanic White, and 96 (60.0%) had a parent with a college degree or more. Manipulative designs promoted prolonged gameplay or purchases through 4 user experience typologies: parasocial relationship pressure occurred in 33 (24.8%) and 25 (18.8%) apps with characters; time pressure in 23 (17.3%) and 14 (10.5%) apps; navigation constraints in 61 (45.9%) and 49 (36.8%) apps; and attractive lures in 60 (45.1%) and 61 (45.9%) apps, respectively. Children from households whose parents had lower education levels had higher manipulative design prevalence scores than children whose parents had graduated from college (median [IQR] 3.7 [2.5-5.0] vs 3.0 [2.0-4.0]; *P* = .02), gameplay-prolonging design (2.3 [1.6-3.0] vs 2.0 [1.5-2.8]; *P* = .047), and purchase pressure (1.0 [0.5-1.5] vs 0.6 [0-1.3]; *P* = .02). Purchase pressure prevalence scores were higher for children from households with lower income (*R* = −0.18; *P* = .02).

**Conclusions and Relevance:**

Design features that encourage monetization of children’s digital experiences were common in this sample and disproportionately occurred in apps used by children with lower SES.

## Key Points

**Question:**

What types of manipulative design features exist in childrens’ mobile apps, and do inequities exist in young childrens exposure to manipulative design?

**Findings:**

In this cross-sectional study of apps used by 160 children aged 3 to 5 years, the majority of apps were associated with manipulative design features that included parasocial relationship pressure, fabricated time pressure, navigation constraints, and use of attractive lures to encourage longer gameplay or more purchases, in addition to advertisement-based pressure; only 20% of apps had no manipulative design features. Children from lower socioeconomic strata played apps with more manipulative design.

**Meaning:**

These results suggest that interactive designs that serve the interests of technology companies over the interests of children are common and deserve further study and regulation.

## Teaser

This cross-sectional study examines manipulative design features of mobile apps intended for children aged 3 to 5 years and the association of prevalence of manipulative features with household socioeconomic status.

## Introduction

Young children are avid users of touchscreen devices, which allow contingent interactivity between users and applications (commonly referred to as *apps*). Despite its many benefits, interactivity has also enabled a host of what design experts call “dark patterns,” interactive designs that benefit the interests of the technology designer (eg, company, website, video game) at the expense of the user.^1,2^ Dark patterns were first described within video games; examples included features that demanded players disrupt their daily activities to return to the game or tedious tasks that could only be avoided by making purchases (termed “grinding”).^1^ Dark patterns are pervasive and have since been identified in many contexts outside of gaming. For example, they are used to exert purchasing pressure on users of e-commerce websites,^3^ encourage excessive labor from workers on gig economy platforms,^4^ and exploit people’s social belonging to increase information disclosure.^5^

A small body of prior work suggests dark patterns are present in interactive apps for children as well. For example, childrens’ apps often monetize gameplay by showing advertisements, encouraging in-app purchases,^6^ or collecting data.^7^ To our knowledge, only one study has attempted to characterize the dark patterns children encounter. Fitton and Read^8^ developed a framework validated through interviews with teenage girls, which included temporal patterns like grinding, monetary patterns like paying for enhancements, and sneaky advertisements that pop up unprompted. However, that analysis did not examine the prevalence of these patterns in childrens’ digital products.

To address these gaps in understanding about age-inappropriate digital design, we leveraged data from a cohort study of young children whose mobile device use was objectively measured. We focused on young children because of their less-developed understanding of persuasive intent (ie, in advertising),^9^ which may render them more susceptible to manipulative designs. This study aimed to (1) develop a reproducible framework for characterizing dark patterns in children’s apps (we refer to these as “manipulative design features” to avoid associating the racialized term “dark” with unethical behavior); (2) describe the frequency of manipulative design in a sample of apps used by young children; (3) examine differences in manipulative design prevalence by app characteristics (ie, by category and free vs paid); and (4) test the hypothesis that children from lower socioeconomic status households, who experience access barriers to high-quality media,^10,11^ would be exposed to apps with more manipulative design features.

## Methods

### Population and Study Design

We analyzed data from the Preschooler Tablet Study, a longitudinal single-center study assessing child development and mobile device use in 3-to-5-year-old children. Data were collected in 3 waves: baseline (phase 1), 3 months (phase 2), and 6 months (phase 3); parents received up to $150 for completion of all phases. The study was approved by the University of Michigan institutional review board, and written informed consent was obtained from parents or legal guardians.

**Figure 1.**
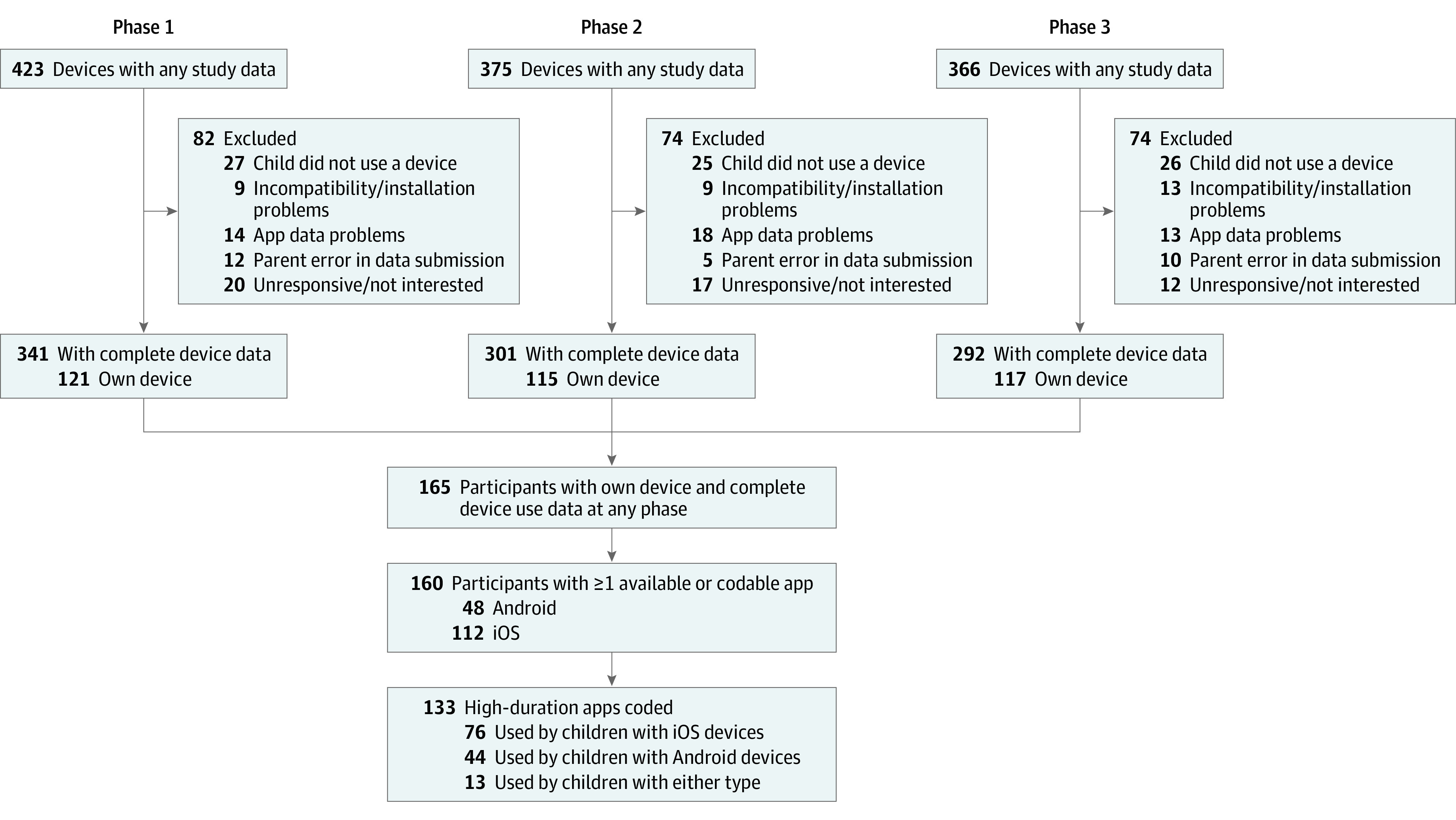
Study Flow Diagram Illustrating Participant Device Data Collection, Missingness, and Apps Selected for Coding

The study population and recruitment methods have been described in detail previously.^12^ This report follows the Strengthening the Reporting of Observational Studies in Epidemiology (STROBE) reporting guideline for observational studies and a study flow diagram shows sample sizes at each phase (Figure 1). A community-based convenience sample of English-speaking parents of 3-to-5-year-old children was recruited; parents or legal guardians were eligible if they lived with the child at least 5 days per week and owned an Android or iOS tablet or smartphone. After providing online consent for themselves and their child, parents were emailed instructions for mobile device sampling and REDCap^13,14^ surveys in which they reported sociodemographic characteristics, including household income and size (from which income-to-needs ratio [ITN] was calculated), and the participating parent’s highest educational attainment. ITN was calculated by dividing annual household income by the US Department of Health and Human Services poverty guideline for the corresponding year and household size. An ITN of 1.0 represents a family living at the federal poverty level; 2.0 represents 200% of the poverty level. In our sample, ITN ranged from 0.15 to 7.39. Parents reported their child’s race and ethnicity; response options were collapsed into non-Hispanic White and minoritized racial and ethnic groups (including Asian or Pacific Islander, Black and African American, Hispanic [any race], multiple races [non-Hispanic], and Native American or Alaska Native) because of small cell sizes for some categories (ie, to increase statistical power) and because of prior research showing differences in media use by child race and ethnicity (Table 1).^10^

**Table 1.** Sociodemographic Characteristics of Preschool-Aged Children With Their Own Mobile Device Participating in the Preschooler Tablet Study

Characteristic	Participants, No. (%)
Child	
Sex	
Girl	70 (43.8)
Boy	90 (56.3)
Age, mean (SD) [range], y	3.96 (0.58) [3.01-5.52]
Race and ethnicity	
Non-Hispanic White	120 (75.0)
Minoritized race or ethnicity^a^	40 (25.0)
Parent	
Sex	
Female	150 (93.8)
Male	10 (6.3)
Age, mean (SD) [range], y	34.5 (4.3) [24.0-45.3]
Marital status	
Married/with partner	145 (90.6)
Single/divorced/separated/widowed	15 (9.4)
Educational attainment	
High school/GED or less	10 (6.3)
Some college or 2-y degree	54 (33.8)
4-y college degree	48 (30.0)
More than 4-y college degree	48 (30.0)
Educational attainment (2-category)	
<4-y college degree	64 (40.0)
≥4-y college degree	96 (60.0)
Employment	
None	47 (29.4)
Part-time	29 (18.1)
Full-time	73 (45.6)
Multiple	11 (6.9)
Child school/child care attendance	
Center-based or home-based	113 (73.9)
Stays home with parent/caregiver	40 (26.1)
Only child	
Yes	33 (20.6)
No	127 (79.4)
Income-to-needs ratio, mean (SD) [range]	3.24 (1.79) [0.15-7.39]

^a^ Minoritized racial and ethnic groups included Asian or Pacific Islander, Black and African American, Hispanic (any race), multiple races (non-Hispanic), and Native American or Alaska Native.

### Mobile Device Sampling

Mobile device sampling occurred at each phase. This method uses data collected by mobile devices to describe childrens’ app usage (for Android devices, the Chronicle app [Methodic] provides timestamped app usage output; for Apple devices, parents collected screenshots of app usage from the phone’s Settings usage archive).^12^ From Chronicle and screenshot output, we generated a list of apps each participant used and the average daily duration of use for each app at all 3 phases. Mobile device data was analyzed using data.table and tidyverse in R versions 3.5.2 through 4.1.1 (R Project for Statistical Computing).

### App Sample for Coding

To create a feasible sample of apps to code, we limited our sample to children who had their own mobile device (165 children), as childrens’ app usage duration could not be calculated on shared devices (ie, parents or siblings might also use high-duration apps such as YouTube, thus skewing daily estimates) (Figure 1). Then, we identified the 3 top-duration apps used by children with their own devices, presuming that manipulative design features would be easier to identify in apps that maintain childrens’ attention for longer. We selected top-duration apps from phase 1 of the study when possible and selected additional top-duration apps from phases 2 or 3 as needed (eAppendix 1 in the Supplement). Children with no available or codable apps were excluded, yielding a sample of 160 children and 133 unique apps; 86 children (53.8%) had 3 codable apps, 46 (28.8%) had 2, and 28 (17.5%) had 1.

### Coding Scheme Development

To develop a manipulative design coding scheme, we used an approach that combined user experience (UX) design concepts,^15^ gambling,^16^ and persuasive design frameworks.^17^ Authors and coders played apps and noted patterns of monetization, behavior of parasocial characters (given their influence on child behavior^18^), reinforcement techniques, and navigability and/or presence of stoppage cues. We refined the coding scheme iteratively through review of codes and app designs with coauthors (A.H., S.C., A.G.).

The resulting framework (Tables 2 and 3) described UX typologies (parasocial pressure, time pressure, navigation constraints, and lures) that appeared designed to achieve monetization goals (ie, prolong gameplay, influence purchase decisions), as well as 3 designs intended to increase advertisement viewing. We defined UX features as manipulative if they used age-inappropriate interaction or reduced user autonomy in service of the monetization goals (eAppendix 2 in the Supplement). Two adult coders trained to reliability (κ = 0.78-0.90) downloaded and played each app for 10 to 15 minutes, completing several levels and visiting every available page (eg, settings, store, level map) to assign apps a binary score for each of 11 possible codes (1 if features present, 0 if not). Coding uncertainties were resolved by consensus (eAppendix 1 in the Supplement).

**Table 2.** Manipulative Design Features to Prolong Gameplay or Encourage Purchases in Apps Played by Preschool-Aged Children

UX typologies and descriptions	To prolong gameplay		To encourage purchases	
	Apps, No. (%) (n = 133)	Children, No. (%) (n = 160)^a^	Apps, No. (%) (n = 133)	Children, No. (%) (n = 160)^a^
Parasocial relationship pressure^b^	33 (24.8)	113 (70.6)	25 (18.8)	106 (66.3)
Time pressure^c^	23 (17.3)	102 (63.8)	14 (10.5)	32 (20.0)
Navigation constraints^d^	61 (45.9)	145 (90.6)	49 (36.8)	63 (39.4)
Lures^e^	60 (45.1)	149 (93.1)	61 (45.9)	69 (43.1)
Total	86 (64.7)	152 (95.0)	74 (55.6)	128 (80.0)

Abbreviation: UX, user experience.

^a^ A manipulative design feature was found in any of the top-duration apps coded for each child.

^b^ App character applies pressure to play longer or make a purchase through shame, taunting, or manipulation.

^c^ Fabricated time pressure at decision points, eg, countdown clock or communication that time is running out; often paired with confusing icons or lures.

^d^ Features constrain where to go or how player orients themselves. May include cumbersome features like frequent pop-up ads; auto-advance without choices to pause or go back; minimization of buttons that avoid purchases or stop gameplay.

^e^ Tokens, rewards, candy, virtual toys, gameplay items, words (eg, “hot item!” “popular choice!”), or visual cues that try to attract attention and encourage behaviors.

**Table 3.** Manipulative Design Features to Encourage Interaction With Advertisements in Apps Played by Preschool-Aged Children

Advertising-related manipulative design descriptions	Frequency in coded apps, No. (%) (n = 133)	Prevalence among 160 children, No. (%) (n = 160)^a^
Roadblock ads^b^	29 (21.8)	46 (28.8)
Strategically timed ads^c^	24 (18.1)	25 (15.6)
Ads with reinforcement^d^	22 (16.5)	25 (15.6)
Any type of ad	42 (31.6)	54 (33.8)

^a^ Refers to whether the manipulative design feature was present in any of the top-duration apps coded for each child.

^b^ Ads appear for more than 20 seconds, pressure the player to play them (sometimes with age-inappropriate or violent content), or navigate player to the app store.

^c^ Ad pops up when the player tries to go back to the home screen (ie, stop playing) or cancels out of a purchase.

^d^ Player is promised gameplay items in exchange for watching ads.

### Statistical Analysis

Across coded apps, we calculated the frequency of each manipulative design feature and created 4 summary scores: (1) gameplay-prolonging features (score range, 0-4); (2) purchase pressure (0-4); (3) advertising pressure (0-3); and (4) sum of all manipulative design features (0-11). For each child, we calculated manipulative design prevalence scores by averaging each summary score across their top apps. Because manipulative design prevalence scores were positively skewed, we conducted Wilcoxon rank-sum tests or Spearman correlations to compare summary scores in free vs paid apps and between app categories (described in prior work^12^), as well as to examine associations between manipulative design prevalence scores and childrens’ socioeconomic characteristics (ITN and parent education). App-level and child-level univariate and bivariate statistics were conducted in SAS version 9.4 (SAS Institute, Inc). Statistical significance was set at *P* < .05 and tests were 2-tailed.

## Results

Of 160 children in the sample, mean (SD) age was 4.0 (0.6) years; 120 children (75.0%) were non-Hispanic White, and 96 (60.0%) had a parent with a college degree or more (Table 1). The eTable in the Supplement shows the names of apps, categories, and manipulative design codes.

### Manipulative UX Typologies

#### Parasocial Relationship Pressure: App Characters or Influencers Behave in Ways That Pressure Users to Prolong Gameplay or Make Purchases

In total, 33 apps (24.8% overall, 29.0% of apps with characters) used parasocial characters to prolong gameplay, either by pressuring the user to keep playing or by expressing disapproval if they stopped. For example, in *My Talking Tom 2*, Talking Tom made statements like, “Do you want to give up?” when the player opted not to move to the next level or “You’re making me want to go to sleep” when the player was idle. Pressure could occur during gameplay (eg, urging player to save friends from a violent farmer in *Green Grandpa Alien*), as a notification to return to the game (eg, *Blockcraft 3D* notifies players that “people are protesting your absence!”), or an enticement to return at a later time (eg, a *DragonML* character saying, “Come back tomorrow to get THIS dragon! Then visit Dragolandia each day for other valuable rewards!”).

Separately, 25 apps (18.8% overall, 21.9% of apps with characters) used parasocial relationships to promote purchases. This pressure sometimes came from a narrator (eg, the narrator of *ABC Animals* says, “You can play with these cute animals for a tiny fee! Ask your parents!”) or a character (eg, the buddy character in *Kick the Buddy* says things like, “Don’t just stand there, buy something!” when the player is on the store page). Video characters on YouTube Kids also encouraged purchases of their branded products. *Todo Math* shows a character with a large tear in their eye as the child is prompted to sign up for a free trial of the paid version.

#### Time-based Pressure: Visual Indicators Conveying Scarcity of Time

Apps displayed countdown clocks and other visual indications of time running out, which is known to interfere with decision-making.^19^ Time pressure was used to prolong gameplay in 23 apps (17.3%) and to promote purchases in 14 apps (10.5%), usually at decision points between levels (eg, *Miraculous* stating, “Save me!” with 4 seconds remaining). Time-based pressure to encourage purchases typically appeared as messaging indicating artificial scarcity (eg, “Limited time only!”) or a countdown indicator that a discount or item would disappear if the user did not act (such as the “Flash deals” advertised in *Subway Surfer*).

#### Navigation Constraints: Obstacles Blocking the User’s Ability to Maneuver Within the Interface

Features like tunneling (providing no options for where to go next), pop-ups, or auto-advancing were used to prolong gameplay in 61 apps (45.9%) and to promote purchases in 49 apps (36.8%). This included making it difficult to reverse or opt out of a level back to the homepage or auto-advancing without providing a clear pause or disengagement options between levels (eg, *Race Master* only provides an option for players to proceed to the next level after finishing a level). In other apps, like *Robocar Poli Habit—Kids Game Package*, the player is not provided with an “X” button but must instead click the “Settings” button, which eventually navigates to the home page.

Navigation constraints to promote purchases appeared in several forms. The most common was asymmetric design that highlighted purchase options and made opt-outs hard to see (eg, lighter “X” and confusingly worded “Restore purchase” buttons in *Kick the Buddy*). Other apps made the in-app store the landing page (eg, *Miraculous*) or had store pages that featured only paid items first, making it cumbersome to find free items. Navigation constraints that promoted purchases also included touchscreen hit pads that promoted purchases (eg, *Sonic Classi*c app had an inaccurately wide “REMOVE ADS” banner) or prompts to purchase items between each level (eg, *Super Jabber Jump 2*).

#### Lures*: *Bringing Attention to an Attractive Object—Including Stickers, Trophies, and Leaderboards—Just as the Player is Trying to Make a Decision

Lures were presented at decision points to prolong gameplay in 60 apps (45.1%) and encourage purchases in 61 apps (45.9%). Lures were coded when they enticed players to engage repeatedly with the app (eg, daily rewards in *Green Grandpa Alien*) or offered as achievements users could earn for repeated play (eg, *DisneyNow* displays types of virtual items players can earn for gameplay targets), sometimes repetitive in nature (eg, *Scribblenauts Remix* offers a gold crown for a repetitive gameplay). Lure purchase pressure usually involved sparkles, pulsing buttons, exclamation points, brightly colored banners that obscure other items, or other attention-grabbing cues on pages where money would be spent (eg, *Kick The Buddy* store page uses visual lures to encourage higher purchase levels).

Advertising-related manipulative designs took 3 forms. First, roadblock advertisements (referred to as *ads*) stayed up for over 20 seconds and prompted the player to interact before allowing the ad to be closed (eg, the ad for *Mr. Bullet* prompted the user to swipe and thereby make Santa shoot people). At times, the “X” to close out the ad was replaced with an “>>” icon, which when clicked would take the user to the app store. These appeared in 29 apps (21.8%). Second, in strategically timed ads (24 apps [18.1%]), ads would pop up when the player chose not to make a purchase or tried to navigate back to the home screen. Third were ads with reinforcement (22 apps [16.5%]), in which in-game rewards were given in exchange for ad viewing, usually providing free lives, new gameplay items, or updated features.

### App-Level Analyses

Apps contained a mean (SD) 3.0 (2.7) manipulative design features (median [IQR], 2 [1-5]; range, 0-10); 26 apps (19.6%) contained none. The most frequent monetization goal was prolonging gameplay, while lures and navigation constraints were the most common UX typologies (Tables 2 and 3). When comparing the 109 free apps analyzed with the 24 paid ones, free apps showed more total manipulative design features (median [IQR] 3.0 [1.0-5.0] vs 1.0 [0-2.0]; *P* < .001), in addition to gameplay (1.0 [0-2.0] vs 0.5 [0-1.5], *P* = .04), purchase (1.0 [0-2.0] vs 0 [0-0.5]; *P* < .001), and advertising (0 [0-1.0] vs 0 [0]; *P* < .001) features.

**Figure 2.**
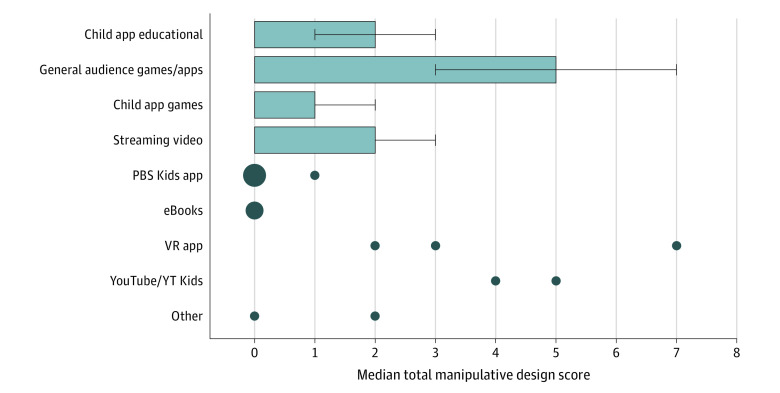
Total Manipulative Design Scores Across Different App Categories Played by Preschool-Aged Children With Their Own Mobile Devices Medians (with IQRs represented in error bars) are shown for app categories with 9 or more apps; individual data points are shown for smaller categories. VR indicates virtual reality; YT, YouTube.

Total manipulative design scores differed significantly between app categories (considering only categories with more than 2 apps, *P* < .001). The highest total manipulative design scores were present in general audience apps, significantly higher than e-books, PBS KIDS apps, educational apps, and early childhood games in Dunn post hoc comparisons (Figure 2).^20^

### Child-Level Analyses

Almost all children (158 [98.8%]) had at least 1 manipulative design feature in their top-duration apps. Compared with children whose parents had graduated from college (96 children), children from lower-education households (64 children) had higher total manipulative design prevalence scores (median [IQR] 3.0 [2-4] vs 3.7 [2.5-5]; *P* = .02), gameplay-prolonging scores (2.0 [1.5-2.8] vs 2.3 [1.6-3]; *P* = .047), and purchase pressure scores (0.6 [0-1.3] vs 1.0 [0.5-1.5]; *P* = .02). Purchase pressure scores were higher for children in lower-income households (*R* = −0.18; *P* = .02), but other manipulative design prevalence scores did not vary with ITN (data not shown).

## Discussion

In this app-content analysis nested within a community-based cohort study, we defined the concept of manipulative design features relative to childrens’ interactive experiences. Manipulative designs were common and clustered into 4 UX typologies—parasocial relationship pressure, time pressure, navigation constraints, and lures—with goals of encouraging gameplay, purchases, and advertisement viewing. These findings contribute to the literature by documenting the high frequency of manipulative designs in apps played by children.

Manipulative design features to prolong gameplay or reengage with the app were the most common design feature in this sample. Apps’ success is typically evaluated based on metrics such as duration and frequency of use,^21,22^ which is likely why app developers use design tricks that advance these goals. The designs we describe in this study reduced user autonomy to pause or end gameplay; applied pressure from favorite characters, to which young children may be particularly susceptible^18^; or provided virtual rewards that appeal to childrens’ immature impulse inhibition.^23,24^ Important next steps will be to investigate how child users respond to these design cues in real-time, whether and how childrens’ ability to transition away from devices changes when engagement-prolonging cues are present, and whether manipulative design leads to displacement of nondigital daily activities.

Manipulative design features to promote purchases occurred most often through navigation constraints and lures. Young children, whose attention may be more attracted to novelty, may be especially susceptible to attention-grabbing features. In addition, children may be less likely than adults to realize that time pressure is fabricated for persuasive purposes.^25^ The US Federal Trade Commission regulates “deceptive financial practice” in general, but more needs to be known about how children interpret interactive nudges to make purchases with real or virtual currency. For these reasons, and reports of children spending thousands of dollars on in-app purchases,^26^ ethical standards are needed regarding purchase-related design features in apps likely to be used by children.

Navigation constraints were another common UX typology used to tunnel players into prolonged gameplay or purchases, but have received less attention in the literature.^1,2,4^ Players may not be aware of how these subtle ways of obscuring navigation options shape their gameplay or decision-making—particularly child users, who need more explicit and transparent design cues to support digital critical thinking skills.^27^

Advertising is highly prevalent in all free digital products, but we identified several features that were more coercive than simple pop-up ads. In 21.8% of apps in this sample, we encountered ads that prompted the player to engage with interactive elements, sometimes with violent content, and with “X” hitpads that navigated the user to the app store. These are novel features compared with our prior analysis of in-app advertising practices,^6^ and they suggest that more regulation of mobile ad networks is needed.

We found that manipulative design features were most common in apps labeled as for “general audiences.” Interestingly, this category of apps had one of the longest daily usage durations in our prior analysis of mobile device data from this cohort,^12^ which raises the possibility that manipulative features are extending children’s time on these apps. In contrast, apps made by the nonprofit group PBS KIDS had zero manipulative design features (other than use of autoplay), which suggests that these apps are more likely to provide positive digital experiences.

Finally, children from low-SES families were more likely to encounter manipulative design features. Although these results are exploratory, they build upon existing evidence of inequities in child access to quality media^10^ by demonstrating an uneven prevalence of manipulative designs intended to monetize childrens’ digital experiences. Replication in other samples and wider age ranges are important future research directions.

### Limitations

Limitations of our study included use of a convenience sample of children with their own mobile devices, whose media use practices may not reflect that of the general population. Our results with respect to socioeconomic status may therefore not be generalizable. We only coded participants’ longest-duration apps, so these results may overestimate the prevalence of manipulative design in childrens’ apps overall.

## Conclusions

This study contributes to child-centered design policy discussions, such as the Age-Appropriate Design Code in the United Kingdom, Digital Services Act in the European Union, and legislation under consideration in the US. Because many digital products are designed by teams without backgrounds in child development, design decisions may be motivated more by monetization and less by how a child experiences their product. There is therefore a pressing need for government, regulatory, or industry actions to ensure that childrens’ needs are considered before digital products are released to market.

## Notes

Supplement. **eAppendix 1.** Coding Approach **eAppendix 2.** Manipulative Design Features Coding Scheme **eTable.** Manipulative Design Features Documented in Apps Played by Preschool-Aged Children, Listed by App Category and Sorted From Highest Total Manipulative Design Score to Lowest
