# Supplementary material for: Prevalence and Characteristics of Manipulative Design in Mobile Applications Used by Children
Source: JAMA Netw Open. 2022 Jun 17;5(6):e2217641. doi: 10.1001/jamanetworkopen.2022.17641 (PMC9206186; doi:10.1001/jamanetworkopen.2022.17641)
Supplement: Supplement. — eAppendix 1. Coding Approach eAppendix 2. Manipulative Design Features Coding Scheme eTable. Manipulative Design Features Documented in Apps Played by Preschool-Aged Children, Listed by App Category and Sorted From Highest Total Manipulative Design Score to Lowest [file jamanetwopen-e2217641-s001.pdf]

Supplemental Online Content

Radesky J, Hiniker A, McLaren C, et al. Prevalence and characteristics of manipulative design in mobile applications used by children. *JAMA Netw Open.* 2022;5(6):e2217641. doi:10.1001/jamanetworkopen.2022.17641

eAppendix 1. Coding Approach

eAppendix 2. Manipulative Design Features Coding Scheme

eTable. Manipulative Design Features Documented in Apps Played by Preschool-Aged Children, Listed by App Category and Sorted From Highest Total Manipulative Design Score to Lowest

This supplemental material has been provided by the authors to give readers additional information about their work.

© 2022 Radesky J et al. *JAMA Network Open.*

eAppendix 1. Coding Approach

Apps were selected based on having the longest duration of average daily use (top 3 per participant). Because we had 3 phases of app usage data, we started by using the top-duration apps from Phase 1. If a participant did not have Phase 1 device data, used less than 3 apps, or any of their top-duration apps were unavailable (i.e., had been removed from the app store) or uncodable (e.g., built-in apps that would not be expected to have manipulative design techniques such as texting, camera, clock, or browser), we selected additional top-duration apps using the same approach from Phase 2 and then Phase 3, in order to get 3 apps for each participant when possible.

Coders were instructed to play each app for about 10-15 minutes, completing several levels (if applicable) and visiting every available page (e.g, settings, store, level map) to observe for design abuses and ease of navigation. If a tutorial was available at the start of the app, coders were instructed to watch it to know how to play the game. Coders switched between apps during gameplay and returned to apps several days later to elicit any design abuses that occur when exiting or notifications prompting the user to return to the app. Coders also were idle for about 60 seconds during gameplay to elicit any reactions from the app regarding restarting playing. Coders additionally navigated to the store section of each app, if present, and took steps to purchase an item, which they cancelled right before confirming the purchase, to see if design abuses occurred in this context. When apps offered in-app items or rewards in exchange for ad viewing, coders watched several ads to describe how this occurred. After accruing in-game currency, they also purchased items (both which they could afford, and ones that were too expensive), to elicit how the app communicated with the player around these transactions. If apps required a free trial, coders created one with our research lab's email and waited through the free trial period to examine what pressure occurred to purchase the app. Coders did not code sections that were labeled as "for parents," as we presumed that these sections would not be designed with child users in mind.

© 2022 Radesky J et al. *JAMA Network Open.*

eAppendix 2. Manipulative Design Features Coding Scheme

**CODE NAME**<br>**Description**
**PARASOCIAL RELATIONSHIP PRESSURE (To prolong gameplay or encourage purchases)**<br>*Code 999 if there are no animated/anthropomorphized characters that respond to your actions. If there are illustrations like on GoNoodle, but they don't interact with you or tell you what to do, then code 999. Some characters may seem really inert/not interactive, but if the child is playing with them in any way, apply a 0 or 1 code.*<br><br>This code describes behavior of a character (animated object, animal, humanoid, etc) in the app that applies pressure to the child to either play longer or make a purchase. It leverages the social reciprocity impulse when children are online. Consider the behavior (verbal, nonverbal) of any character, written statements on the screen, or voice-over/narrator communication with you while you play. Even a smiley face on a glass in Happy Glass counts as a character, especially if it responds to your actions.<br><br>Behavior is considered abusive if it shames the player, seems manipulative (e.g., the character is crying or hurt, and will only be relieved if the child keeps playing.) Taunting, insults, or pressure to rescue characters in the game are included. Character may verbally urge you to keep playing or point to the play button.<br><br>Examples that are NOT Parasocial Relationship Abuse: Sonic looking at his watch and tapping his foot when the player is idle, since he is not showing overt disapproval; My Baby Unicorn showing a thought bubble of what the player should do next and showing related behaviors (e.g., drooling and thinking of food), because the unicorn is not shown to suffer in any way. The Subway Surfer character does different moves on the page when you finish a level, but he doesn't explicitly point to any of the buttons to encourage the player to click them.
**TIME PRESSURE (To prolong gameplay or encourage purchases)**<br>This code describes when time pressure is present, such as a visible countdown clock or verbal/written communication that time is running out. Usually linked with a free life, discounts on purchasing, or extra coins/token. Feels like in-the-moment contrived pressure to click on something, similar to artificial scarcity in e-commerce sites. May be accompanied by confusing icons (e.g., to add cognitive load during decision making)<br><br>Note: This does NOT include the timers that indicate how many minutes/hours later the user should come back LATER to get free coins or other rewards (see Lures)

© 2022 Radesky J et al. *JAMA Network Open.*

**NAVIGATION CONSTRAINTS (To prolong gameplay or encourage purchases)**<br>These are UX features that constrain where you can go, or how easy it is to navigate around the game or know where you are/orient yourself. Examples of easy navigability include the app having a home screen that can always be accessed through a clearly marked button, a map that shows where you are relative to other levels, and easy “back” options that let you recover if you make the wrong decision.<br><br>Designs that contribute to worse navigability include lots of pop-up ads that get in the way of going to the next level or home screen; auto-advance without choices to pause or go back; minimization of buttons that avoid purchases or stop gameplay. You might get a sense of disorientation in the game that may result from lack of symmetry of UI features. It’s unclear what will happen when buttons are clicked.<br><br>Note: it’s OK if the app gives you a pre-determined # of free days or plays, and is clear with you about how many you have left. Also OK to have parent gate before purchases. These are appropriate navigation constraints, but if there are other manipulative features in addition to these, code a 1.
**LURES (To prolong gameplay or encourage purchases)**<br>Lures are tokens, rewards, candy, virtual toys, gameplay items, words (e.g., “hot item!” “popular choice!”) or visual cues that try to attract attention and encourage behaviors (gameplay, purchases, clicking on ads). They can be present anywhere in the game experience, but often are at the end of a level (sometimes with a countdown timer), in the store/purchase area, or on the homepage.
**INTERACTION WITH ADVERTISEMENTS**<br>**ROADBLOCK ADS** exist when the ads force the player to watch them (stay up >20 sec with no “x”), prompt user to play them (sometimes with age-inappropriate or violent content), or navigate player to the app store.<br><br>**STRATEGICALLY TIMED ADS** occur when an ad pops up when the player tries to go back to the home screen (i.e., stop playing) or X’s out of a purchase – akin to a punishment for choosing to disengage.<br><br>**ADS WITH REINFORCEMENT** occur when the player is promised gameplay items, tokens, or rewards for watching ads.

© 2022 Radesky J et al. *JAMA Network Open.*

eTable. Manipulative Design Features Documented in Apps Played by Preschool-Aged Children, Listed by App Category and Sorted From Highest Total Manipulative Design Score to Lowest

Android Common name (app package name)	iOS app name*	N users**	Parasocial relationship pressure		Time pressure		Navigation constraints		Lures		Roadblock ads	Strategically timed ads	Ads with reinforcement	Total manipulative design score
			GP	$	GP	$	GP	$	GP	$				
**Early Childhood Apps Labeled "Educational"**														
ABC Mouse (mobi.abcmouse.academy_goo)	ABCmouse	8	1	1	0	1	1	1	1	1	0	0	0	7
Poli Habit Game (com.kigle.project.poli.habit)		1	1	0	0	0	1	1	1	1	1	0	0	6
	KidsDoodle	1	0	0	0	0	1	1	0	1	1	1	1	6
	TodoMath	1	1	1	0	1	0	1	1	1	0	0	0	6
	Epic!	1	1	1	0	0	0	1	1	1	0	0	0	5
My Baby Unicorn (com.tutotoons.app.mybabyunicorn.free)		1	0	0	0	0	0	1	0	0	1	1	1	4
	Academy	1	1	0	1	0	0	0	1	1	0	0	0	4
	Air & Sea	1	1	0	0	0	0	1	1	1	0	0	0	4
	World	1	0	1	0	1	0	1	0	1	0	0	0	4
Car Game for Toddlers (com.amayasoft.cars.kids.toddlers.garage.game)		1	0	1	0	0	0	1	0	1	0	0	0	3
Wheels on the Bus (com.duckduckmoosedesign.bus)	WheelsOnBus	2	0	0	0	0	1	0	0	1	1	0	0	3
	ABC Animals	1	0	1	0	0	0	1	0	1	0	0	0	3
	Cubic Frog	1	0	0	0	0	1	1	0	1	0	0	0	3
	Fix It!	1	1	0	0	0	1	0	1	0	0	0	0	3
	Learning	1	0	1	0	0	0	1	0	1	0	0	0	3
	Reader	1	0	0	0	0	1	1	0	1	0	0	0	3
	Teach Monster	3	1	0	0	0	1	0	1	0	0	0	0	3
Toddler Games for 2-5 Year Olds (com.bimiboo.playandlearn)		1	0	0	0	0	0	1	0	1	0	0	0	2
Kids Learn to Count 123 (com.intellijoy.counting)		1	0	0	0	0	0	1	0	1	0	0	0	2
Christmas Coloring Book (com.orange.coloring.book.christmas.kids)		1	999	999	0	0	0	0	0	0	1	1	0	2
Very Hungry Caterpillar (com.storytoys.myveryhungrycaterpillar.free.android.googleplay)		1	0	0	0	0	0	1	0	1	0	0	0	2
Sight Words Learning Games & Flash Cards Lite (cz.cfc.androidgp.sightwordslite)		1	0	0	0	0	0	1	1	0	0	0	0	2
	Elmo ABCs Lite	1	1	0	0	0	1	0	0	0	0	0	0	2
	Endless ABC	1	0	0	0	0	1	1	0	0	0	0	0	2
	NOGGIN	2	1	0	0	0	0	1	0	0	0	0	0	2

© 2022 Radesky J et al. *JAMA Network Open.*

	Osmo	1	999	999	0	0	1	0	1	0	0	0	0	2
	Peppa Pig Holiday	1	0	0	0	0	1	0	1	0	0	0	0	2
	Preschool	2	0	0	0	0	0	1	0	1	0	0	0	2
	Race Master	1	999	999	0	0	1	0	0	0	1	0	0	2
Fish School (com.duckduckmoosedesign.fish)		1	0	0	0	0	1	0	0	0	0	0	0	1
	123 Learn	1	0	0	0	0	0	0	0	1	0	0	0	1
	Elmo 123s Lite	1	0	0	0	0	1	0	0	0	0	0	0	1
	G In Space	1	0	1	0	0	0	0	0	0	0	0	0	1
	Kiddopia	3	0	0	0	0	0	1	0	0	0	0	0	1
	Lunchbox	1	0	0	0	0	1	0	0	0	0	0	0	1
	Sorter	1	0	0	0	0	0	0	0	0	0	1	0	1
	Starfall ABCs	1	999	999	0	0	0	0	0	1	0	0	0	1
	StoryLineOnline	1	0	0	1	0	0	0	0	0	0	0	0	1
Elephant Learning Math Academy (com.elephanthead.elephantlearning)		1	0	0	0	0	0	0	0	0	0	0	0	0
Puzzle Kids (com.rvappstudios.jigsaw.puzzles.kids)		1	0	0	0	0	0	0	0	0	0	0	0	0
Elmo Calls by Sesame Street (com.sesame.apps.elmocalls.android)		1	0	0	0	0	0	0	0	0	0	0	0	0
Baby Panda's Treasure Island (com.sinyee.babybus.digging)		1	0	0	0	0	0	0	0	0	0	0	0	0
Khan Academy Kids (org.khankids.android)		2	0	0	0	0	0	0	0	0	0	0	0	0
	Baby Blocks	1	0	0	0	0	0	0	0	0	0	0	0	0
	Endless 123	1	0	0	0	0	0	0	0	0	0	0	0	0
	Learning Games	2	0	0	0	0	0	0	0	0	0	0	0	0
	Reading	1	0	0	0	0	0	0	0	0	0	0	0	0
	Sesame Street	1	0	0	0	0	0	0	0	0	0	0	0	0
	Tozzle	1	0	0	0	0	0	0	0	0	0	0	0	0
**Early Childhood Games (not labeled "Educational")**														
Animal Crossing: Pocket Camp (com.nintendo.zaca)		1	1	1	1	1	1	1	1	1	0	0	0	8
	Kids' Puzzles	1	999	999	1	1	0	1	1	1	0	0	1	6
	MagicTracks	1	0	0	0	0	1	0	1	1	0	0	0	3
Repair Machines – Monster Trucks (com.YovoGames.carRepair3)		1	0	0	0	0	0	1	0	0	0	1	0	2
	Magic Timer	1	0	0	0	0	0	0	1	1	0	0	0	2
	DinoDigger	1	0	0	0	0	0	1	0	0	0	0	0	1
	Holiday Trucks and Diggers	1	0	0	0	0	1	0	0	0	0	0	0	1
	Love2Learn	1	0	0	0	0	1	0	0	0	0	0	0	1
	Monster 2	1	1	0	0	0	0	0	0	0	0	0	0	1

© 2022 Radesky J et al. *JAMA Network Open.*

	Princess	1	999	999	0	0	0	0	0	0	1	0	0	1
	Road Trip	1	0	0	0	0	1	0	0	0	0	0	0	1
	Robot Lab	1	0	0	0	0	1	0	0	0	0	0	0	1
Toca Mini (com.tocaboca.fuhu.tocamini)		1	0	0	0	0	0	0	0	0	0	0	0	0
Toca Nature (com.tocaboca.tocanature)		1	0	0	0	0	0	0	0	0	0	0	0	0
	Toca Stable	1	0	0	0	0	0	0	0	0	0	0	0	0
**Streaming Video Platforms**														
Amazon Free Time (com.amazon.tahoe)		2	0	0	0	0	1	1	1	0	0	0	0	3
DisneyNOW (com.disney.datg.videoplatforms.android.watchdc)	DisneyNOW	6	0	0	1	0	0	0	1	0	1	0	0	3
Netflix (com.netflix.mediaclient)	Netflix	51	999	999	1	0	1	0	1	0	0	0	0	3
	Appisodes	2	0	0	1	0	0	0	1	0	1	0	0	3
Prime Video (com.amazon.avod.thirdpartyclient)	Prime Video	13	999	999	0	0	1	0	1	0	0	0	0	2
	GoNoodle Kids	1	999	999	0	0	1	0	0	0	1	0	0	2
	Hulu	4	999	999	1	0	0	0	0	1	0	0	0	2
Nick Jr. (com.nick.android.nickjr)	Nick Jr.	11	0	0	0	0	0	0	0	0	1	0	0	1
Living Scriptures (com.alldigital.android.livingscriptures)		1	999	999	0	0	0	0	0	0	0	0	0	0
**YouTube/YouTube Kids**														
YouTube (com.google.android.youtube)	YouTube	40	1	1	1	0	1	0	1	0	0	0	0	5
YouTube Kids (com.google.android.apps.youtube.kids)	YT Kids	64	1	1	0	0	1	0	1	0	0	0	0	4
**General Audience Games/Apps**														
	Tom Gold Run	3	1	0	1	1	1	1	1	1	1	1	1	10
Dragon Mania Legends – Animal Fantasy (com.gameloft.android.ANMP.GloftDOHM)		1	1	1	1	1	1	1	1	1	0	0	1	9
Temple Run 2 (com.imangi.templerun2)		1	1	0	1	0	1	1	1	1	1	1	1	9
Green Grandpa Alien (com.scarydudes.green.grandpa.alien)		1	1	1	1	1	0	1	1	1	0	1	1	9
	Happy Glass	2	1	0	1	1	0	1	1	1	1	1	1	9
Miraculous Ladybug & Cat Noir – The Official Game (com.crazylabs.lady.bug)		1	1	0	1	0	1	1	1	1	1	0	1	8
Block Craft 3D (com.fungames.blockcraft)	Block Craft 3D	3	1	0	0	0	1	1	1	1	1	1	1	8
My Talking Tom 2 (com.outfit7.mytalkingtom2)		1	1	1	0	0	1	0	1	1	1	1	1	8
	Extreme Car Driving Simulator	1	0	0	1	0	1	1	1	1	1	1	1	8
	Subway Surf	3	0	0	1	1	1	1	1	1	1	0	1	8
Plants vs. Zombies 2 FREE (com.ea.game.pvz2_na)		1	1	0	0	1	1	1	1	1	0	0	1	7
Super Jabber Jump 2 (com.gameone.jabbersuperjump2.free)		1	0	0	1	0	1	1	1	1	0	1	1	7
Angry Birds 2 (com.rovio.baba)		1	1	1	1	0	1	0	1	1	0	1	0	7

© 2022 Radesky J et al. *JAMA Network Open.*

	Dance Clash	1	0	1	0	0	1	0	1	1	1	1	1	7
	Despicable	1	1	1	0	0	1	1	1	1	1	0	0	7
	Helix Jump	1	999	999	1	0	1	1	1	1	1	0	1	7
Strawberry Shortcake Ice Cream Island (com.budgestudios.googleplay.StrawberryShortcakeIceCreamIsland)		1	1	1	0	0	0	1	1	1	0	1	0	6
Kick the Buddy (com.playgendary.kickthebuddy)	KickTheBuddy	2	1	1	0	0	1	1	1	1	0	0	0	6
	Block City Wars	1	1	0	0	0	1	0	1	1	1	0	1	6
	Sausage Run	1	0	0	1	0	1	0	1	0	1	1	1	6
My Singing Monsters (com.bigbluebubble.singingmonsters.full)		1	0	1	0	1	1	0	1	1	0	0	0	5
Rider (com.ketchapp.rider)		1	999	999	0	0	0	0	1	1	1	1	1	5
Talking Pierre the Parrot (com.outfit7.talkingpierrefree)		1	0	0	1	0	1	1	0	1	0	1	0	5
Roblox (com.roblox.client)	Roblox	10	0	0	0	1	1	1	1	1	0	0	0	5
Sonic the Hedgehog 2 Classic (com.sega.sonic2.runner)		1	0	0	0	0	0	1	1	1	1	1	0	5
CATS: Crash Arena Turbo Stars (com.zeptolab.cats.google)		1	1	1	0	0	1	1	0	1	0	0	0	5
	Candy Crush Saga	1	0	0	0	1	1	0	1	1	0	0	1	5
	Dreamhouse	1	1	1	0	0	0	1	1	1	0	0	0	5
	Fishdom	1	0	0	0	0	1	0	1	1	1	1	0	5
	BallPaint	1	0	0	0	0	1	0	1	0	1	1	0	4
	My Bowling 3D	1	999	999	0	0	1	0	1	0	0	1	1	4
WWE Champions 2019 (com.scopely.whiplash)		1	1	0	0	0	1	0	0	1	0	0	0	3
TikTok (com.zhiliaoapp.musically)***		1	0	1	0	0	1	0	1	0	0	0	0	3
	Minecraft	4	1	0	0	0	0	0	1	1	0	0	0	3
Sonic CD Classic (com.sega.soniccd.classic)		1	0	0	0	0	0	1	0	1	0	0	0	2
Scribblenauts Remix (com.wb.goog.scribbleremix)		1	0	0	0	0	0	0	1	1	0	0	0	2
	Happy Color	2	0	0	0	0	0	0	1	1	0	0	0	2
	Terraria	1	0	0	0	0	1	0	1	0	0	0	0	2
	ZombieRT	1	0	0	0	0	1	0	0	1	0	0	0	2
Monoment Valley 2 (com.ustwo.monumentvalley2)		1	0	0	0	0	1	0	0	0	0	0	0	1
	Board Kings	1	0	0	0	0	1	0	0	0	0	0	0	1
	SSG	1	0	0	0	0	0	0	1	0	0	0	0	1
Toca City (com.tocaboca.tocacity)		1	0	0	0	0	0	0	0	0	0	0	0	0
**Augmented Reality / Virtual Reality Apps**														
Thomas & Friends Minis (com.budgestudios.googleplay.ThomasAndFriendsMinis)		1	1	1	0	0	1	1	1	1	0	1	0	7

© 2022 Radesky J et al. *JAMA Network Open.*

Pokemon Go (com.nianticlabs.pokemongo)		1	0	1	0	0	0	0	1	1	0	0	0	3
	Build a house	1	0	0	0	0	0	1	0	0	1	0	0	2
**Other Categories**														
Facebook (com.facebook.katana)	Facebook	3	999	999	0	0	1	0	1	0	0	0	0	2
Gospel Library (org.lds.ldssa)		1	999	999	0	0	0	0	0	0	0	0	0	0
**PBS KIDS Apps**														
	Watch Videos	11	999	999	1	0	0	0	0	0	0	0	0	1
PBS KIDS Games (org.pbskids.gamesapp)	Play Games	12	0	0	0	0	0	0	0	0	0	0	0	0
	Breathe	1	0	0	0	0	0	0	0	0	0	0	0	0
	Daniel Tiger	1	0	0	0	0	0	0	0	0	0	0	0	0
	Explore	1	0	0	0	0	0	0	0	0	0	0	0	0
	Day & Night	2	0	0	0	0	0	0	0	0	0	0	0	0
	Grr-ific Feelings	1	0	0	0	0	0	0	0	0	0	0	0	0
**Electronic books / Reading apps**														
JW Library (org.jw.jwlibrary.mobile)		1	999	999	0	0	0	0	0	0	0	0	0	0
	GrowUp	1	0	0	0	0	0	0	0	0	0	0	0	0
	Kindle	1	999	999	0	0	0	0	0	0	0	0	0	0

GP = To prolong gameplay; $ = To encourage purchases; * App name and screen shot appearance were used to identify specific apps for iOS devices; ** The number of users who had this app in their 3 top-duration apps; 999 indicates apps that did not have interactive characters and therefore were not coded for Parasocial Relationship Pressure; *** When data were collected (2018), TikTok was primarily for viewing music/dance so was not coded as an adult app

© 2022 Radesky J et al. *JAMA Network Open.*
